# Sequential invitations to FOBT screening and colorectal cancer incidence

**DOI:** 10.1038/s41598-026-45674-z

**Published:** 2026-04-18

**Authors:** Xiaoqin Wang, Hanna Ribbing Wilén, Rachael V. Phillips, Zeyi Wang, Mark J. van der Laan, Li Yin, Johannes Blom

**Affiliations:** 1 Department of Electrical Engineering, Mathematics and Science, University of Gävle, Gävle, Sweden; 2 Department of Emergency Surgery, Karolinska University Hospital Huddinge, Stockholm, Sweden; 3 Department of Clinical Science and Education, Karolinska Institutet, Stockholm, Sweden; 4 Division of Biostatistics, School of Public Health, University of California at Berkeley, Berkeley, CA USA; 5 Department of Statistics, Oklahoma State University, Stillwater, OK USA; 6 Department of Medical Epidemiology and Biostatistics, Karolinska Institutet, Stockholm, Sweden; 7 Department of Surgery, Södersjukhuset, Stockholm, Sweden

**Keywords:** Cancer, Diseases, Gastroenterology, Medical research, Oncology, Risk factors

## Abstract

The effect of different sequences of invitations to Faecal Occult Blood Test (FOBT) screening regarding colorectal cancer (CRC) incidence has never been evaluated. In 2008–2012, all residents in Stockholm-Gotland, Sweden, born 1938–1954, were randomly assigned by birth year to different calendar years of invitation to guaiac-based FOBT (g) or Faecal Immunochemical Test (f) screening at 60–69 years (1–5 rounds), or not (0). Linkage was made to the national Cancer- and Cause of Death Registers on CRC diagnosis and mortality 1958–2020, and the Swedish Colorectal Cancer Register regarding stage. Follow-up started age 60 and CRC incidence, calculated per 100,000 person-years, was assessed during screening (age 60–69) and post screening (age 70–73). Stage I-II and III-IV was assessed post screening. 364,668 individuals were included. During screening, incidence rate ratio was significantly higher in sequences (0, g, g, g, g) (RR 1.25, 95% CI 1.09–1.43), (g, g, g, g, f) (RR 1.17, 95% CI 1.01–1.35), and (g, g, f, f, f) (RR 1.14, 95% CI 1.01–1.29). Post screening, the largest decrease was seen in sequences (g, g, g, g, f) and (g, g, g, f, f), RR 0.65, 95%, CI 0.47–0.90, and RR 0.53, 95% CI 0.30–0.94, respectively. There was an overall decreasing trend along sequences from (0, 0, 0, 0, g) to (g, g, f, f, f) post screening and both stages I-II and III-IV (p < 0.001). We could demonstrate a decreased CRC incidence post screening proportional to the number of invitations with implications for future modeling studies and risk-based screening strategies.

**Supplementary Information:**

The online version contains supplementary material available at 10.1038/s41598-026-45674-z.

## Introduction

The primary purpose of colorectal cancer (CRC) screening is to detect cancer at an early curable stage. Both larger precursors – adenomatous polyps (adenomas) – and cancer may bleed, which makes it possible to measure the presence of invisible (occult) blood in the stool with fecal occult blood tests (FOBT) before cancer symptoms develops. As removal of adenomas has a protective effect against the development of CRC, screening programs that detect and treat adenomas have the potential to reduce both CRC incidence and mortality from the disease^1,2^.

The initial screening FOBT was guaiac-based (gFOBT), which, when used biennially, has demonstrated an approximate 15% reduction of CRC mortality in randomized screening trials and in routine screening^1,2^. Fecal immunochemical tests (FIT), that specifically and quantitatively measure human blood in the stool, has a higher sensitivity for adenomas and cancer than gFOBT, and has commonly replaced gFOBT in ongoing screening programs since it is expected to be at least as effective^3,4^.

The European Commission issued recommendations to screen the population 50–74 years old with FOBT in 2003^5^. The recommendations were updated in the 2010 European Guidelines for Quality Assurance in CRC screening, and in 2022, when the European Commission recommended FIT as a triage screening test for referral of individuals aged 50–74 to follow-up colonoscopy^6,7^. Numerous countries around the world are now offering FOBT screening to the average risk population^8^.

Nevertheless, evaluating the effectiveness of a screening program on CRC incidence is challenging. On one hand, during program implementation, an increase of CRC incidence is expected, as the test aims to detect prevalent CRCs^11,13^. On the other hand, the protective effect of the development of CRC with adenoma removal would lead to a long-term decrease of incident CRCs. With a screening program consisting of a sequence of tests, the CRC incidence arises from a highly complex stochastic process induced by the program. This process leads to several complications, including the presence of adenomatous polyps as intermediate variables between the screening invitation and non-compliance with testing, which complicates statistical analyses^11,13–17^.

The aim of this study was to estimate the effect of different sequential schemes of invitation to FOBT screening in Stockholm-Gotland, Sweden, regarding incidence and stage, during and after the screening period. Our study mimics a randomized trial by a random assignment of invitation sequences by birth years to achieve unbiased evaluation of the dynamics of the CRC incidence and stage during and after the screening period and forms a basis for individualized screening programs.

## Material and methods

### The screening program

In 2008, the Region of Stockholm-Gotland, Sweden, started to implement a CRC screening program^9^. When fully expanded, the program biennially invited all residents 60–69 years old, to start screening at the age of 60, *i.e*., five screening rounds. The invitation included a test-kit with three gFOBT samples (Hemoccult, Beckman Coulter, Indianapolis, U.S.A.), instructions on how to perform the test, information on CRC screening, and a prepaid return envelope^10^. In the case of non-response, a reminder was sent after eight weeks. Individuals with a negative test result (three negative samples) were informed by letter and was re-invited in two years. Individuals with a positive test (at least one out of three samples positive) were referred to the endoscopy clinic responsible for assessment of positive individuals in the catchment area, to schedule a colonoscopy within two weeks. If a CRC or advanced adenomatous polyps (adenomas ≥ 10 mm, high-grade dysplasia or villous histology, serrated polyps with dysplasia or ≥ 10 mm in size or ≥ 3 low-risk adenomas) were detected, individuals were referred for surgery or an adenoma surveillance program. Otherwise, when false positive FOBT, they returned to the screening program. In October  2015, the gFOBT was replaced by FIT (OC-Sensor, Eiken Chemical Co, Tokyo, Japan) with a cut-off level of 40 ug Hb/g feces for a positive test in women and 80 ug/g in men^11^.

### Study design

The study was performed on a cohort of approximately 400,000 individuals, who resided in the Region of Stockholm-Gotland in 2008–2012 and was born between 1938 and 1954. No exclusions. The individuals were randomized by birth years to different calendar years of start of invitation to screening or not, with 2008 as the first year and 2015 as the last year to start screning.

As a result, the total number of screening invitations differed according to the age at first invitation. There was a total of 17 birth year cohorts with different ages at first invitation and different sequences of invitation to gFOBT (g), FIT (f) or no invitation (0) over the 10-year screening period from 60 to 69 years of age (Table 1). Three of the sequences were control sequences (birth cohorts 1938, 1939 and 1941), in which no invitation was made throughout the 10-year screening period. The sequences were denoted by (var1, var2, var3, var4, and var5), where var1 represented invitation at 60 years of age, …, and var5 at 68, each variable taking the value of either g, f or 0. Thus (0, 0, 0, 0, 0) is the control sequence corresponding to the non-invited.

**Table 1:**
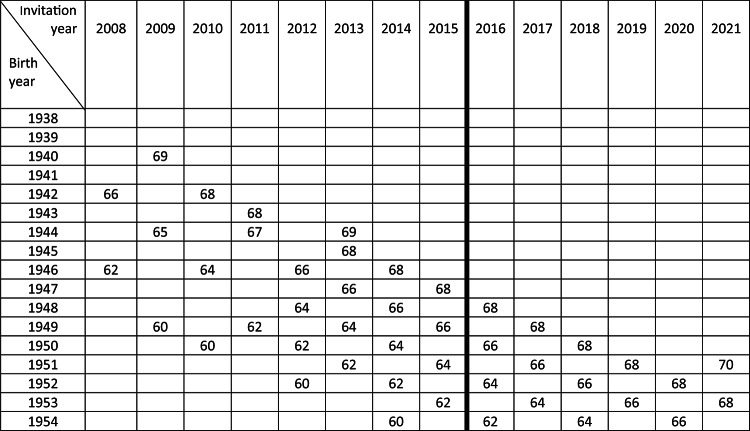
Invitation scheme to the Stockholm-Gotland FOBT screening program by birth year and invitation year. The vertical line marks the shift from gFOBT to FIT in October 2015. Because of short follow-ups, we exclude birth cohorts 1953 and 1954 for the screening-period study (60–69) and additional 1951 and 1952 for the post-screening-period study (70–73).

Consequently, we have CRC incidence and stage as outcomes and invitation sequences as exposures like intention-to-treat as exposure in randomized trials. All individuals started the follow-up at the age of 60 and ended the follow-up at the date of a CRC diagnosis, emigration, death, or on December 31, 2020, whichever occurred first. With start of invitations in 2008, the maximum follow-up was 13 years. The overall compliance to the program was 63.3%, and has been previously reported^12^.

Since all sequences started at age 60, we did not need to adjust for age. Due to our study design, we did not need to adjust for the testing results such as adenomatous polyps or non-compliance to the testing. Furthermore, because all invitation sequences started in close calendar years, other potential CRC risk factors prior to 60 were evenly distributed in different invitation sequences and not needed to be adjusted for. Therefore, we only dealt with gender and calendar year.

### Data retrieval and follow-up

By using the individually unique national registration number assigned to all Swedish residents, the data on cohort individuals, including information on emigration, were retrieved from Statistics Sweden. The individuals were linked to the Cancer Register and the Cause of Death Register at the National Board of Health and Welfare to retrieve information on mortalities during follow-up and CRC diagnosis from 1958 to 2020^13,14^. The CRC diagnoses were coded according to the International Classification of Diseases 7 (ICD-7) as 153.X (malignant neoplasm of large intestine) or 154.0 (malignant neoplasm of rectum), excluding the codes C24; 091 (neuroendocrine tumor), 093 (lymphoma), 094 (adenoma), 144 (squamous cell carcinoma) and 793 (gastrointestinal stroma tumor). Information on CRC stage was retrieved from the Swedish Colorectal Cancer Register (SCRCR), classified according to the TNM system in stage I-IV: stage I (T1-2, N0, M0), stage II (T3-4, N0, M0), stage III (T1-4, N1-2, M0) and stage IV (M1). Early CRC was defined as stage I-II and late CRC as stage as III-IV.

The study was approved by the Swedish Ethical Review Authority (No. 020-06757) and performed in accordance with the Declaration of Helsinki. Informed consent was waived, when all individual data were pseudonymized with code key at Statistics Sweden. All methods were conducted and reported following the Strengthening the Reporting of Observational Studies in Epidemiology (STROBE) Checklist for cohort studies (Supplementary file 1).

### Statistical analyses

We conducted sequential analysis, in which we compared the CRC incidence and stage for different sequences of invitation.

Two follow-up periods were evaluated: between 60 and 69 years of age (screening period), and from 70 to 73 years of age (post screening period). During the screening period, the maximum follow-up was 10 years. During the post screening period, the maximum follow-up was 3 years. We excluded sequences (g, g, f, f, f) and (g, f, f, f, ?), with “?” indicating unknown invitation, because follow-up was too short for individuals to reach 70 years of age by 2020. As the CRC stage information was only available from 2008 and onwards, we conducted the stage analysis only for the post screening period.

Regarding the CRC incidence, the effectiveness of the screening program was measured as the rate ratio (RR) of the invitation sequence relative to the control sequence (non-invited), where rate was defined as the number of incident CRCs divided by the person-years during the follow-up of the sequence. The RRs with 95% confidence intervals (CI) were then estimated using Poisson regression.

CRC stage was categorized into three levels: no CRC, stage I-II and stage III-IV. The stage rate was defined as the number of stage I-II (or III-IV) divided by the person-years during the follow-up of the invitation sequence. The effectiveness of the screening program on stage was measured as the stage rate ratio (RR) of the invitation sequence relative to the control sequence. Because CRC and, hence, different stages of CRC, are rare events in the population, stage rates are well approximated by odds, hence multinomial logistic regression was employed to estimate stage rate ratios. To deal with missing stages, the multiple imputation technique was applied using the multinomial regression conditional on gender and the invitation sequence, where the missing at random is a reasonable assumption conditional on gender and the invitation sequence.

The trend of CRC incidence and stage was analyzed using the ordinary trend test with a linear regression of y on x, where y is the log rate ratio as a continuous variable and x is an ordinal variable with the sequences from low to high, with one unit between each x.

Even if gender is not a confounder according to our study design, we still included it in the above statistical modeling. Furthermore, we needed to address the influence of the calendar year as potential confounder. Because different invitation sequences compiled different calendar years, it was not possible to make adjustment for the calendar year. On the other hand, the calendar year was expected to have minimal influence on CRC risk and stage across the different invitation sequences. In a sensitivity analysis, we used the Swedish population as reference, *i.e.*, the CRC incidence rates at different calendar years in the Swedish male and female populations.

## Results

There were 392,499 residents born 1938–1954 in the region of Stockholm-Gotland, Sweden, in 2008–2012. Thirty-four individuals were excluded because they had multiple first invitation and were missing subsequent invitations, 1,931 were excluded due to emigration before the age of 60, 2,880 individuals died before the age of 60, and 1,775 were excluded due to CRC diagnosis before 60 years of age. Moreover, 21,245 were excluded due to not belonging to any of the groups defined (by birth year, starting calendar year). In total, 364,668 individuals were included in the study (93% of the source population), as described in detail in Table 2.

**Table 2:** Birth cohort sizes, calendar year, age at screening start, and the sequence of invitations to routine FOBT screening in Stockholm–Gotland, Sweden 2008 to 2015.

Birth year	Screening start year	Age at start	Population	Invitation sequence^a^
1938	Non-invited	–	15,368	(0, 0, 0, 0, 0)
1939	Non-invited	–	16,346	(0, 0, 0, 0, 0)
1940	2009	69	15,533	(0, 0, 0, 0, g)
1941	Non-invited	–	18,445	(0, 0, 0, 0, 0)
1942	2008	66	19,956	(0, 0, 0, g, g)
1943	2011	68	22,069	(0, 0, 0, 0, g)
1944	2009	65	24,206	(0, 0, g, g, g)
1945	2013	68	23,879	(0, 0, 0, 0, g)
1946	2008	62	24,742	(0, g, g, g, g)
1947	2013	66	24,041	(0, 0, 0, g, g)
1948	2012	64	24,019	(0, 0, g, g, f)
1949	2009	60	23,208	(g, g, g, g, f)
1950	2010	60	23,305	(g, g, g, f, f)
1951	2013	62	21,825	(g, g, f, f, f)
1952	2012	60	22,893	(g, g, f, f, f)
1953	2015	62	22,268	(g, f, f, f, ?)
1954	2014	60	22,565	(g, f, f, f, ?)

^a^0 = no invitation; g = invitation to guaiac-based fecal occult blood test (gFOBT); f = invitation to fecal immunochemistry test (FIT).

**Fig. 1:**
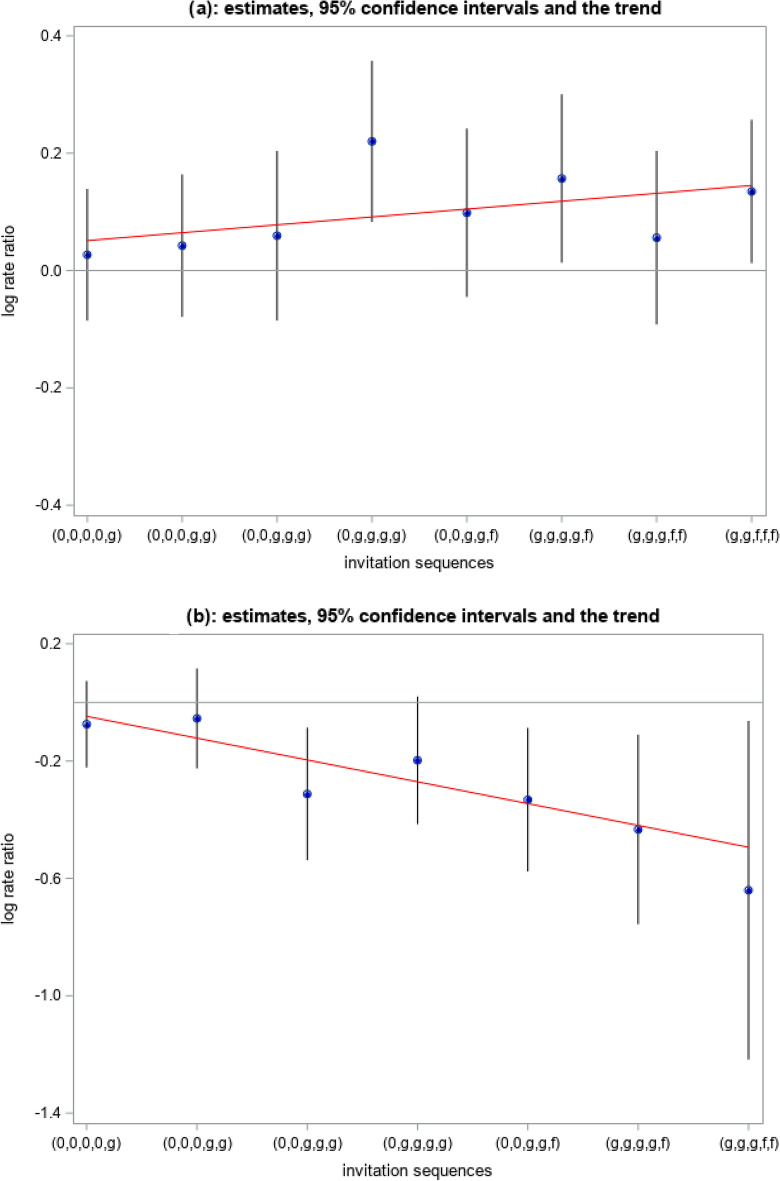
Trend for CRC incidence by individual sequence of invitation to FOBT-screening of the 60–69-year-old population relative to control sequence (non-invited). (**a**) Follow-up between the age of 60 and 69, p-value for trend = 0.085. (**b**) Follow-up between 70 and 73, p-value for trend < 0.001.

As seen in Table 3, all different sequences of invitation to screening between 60 and 69 years of age had an increased CRC incidence rate ratio, as compared to the non-invited. The rate ratio was significantly higher in the sequences (0, g, g, g, g) (RR 1.25, 95% CI 1.09–1.43), (g, g, g, g, f) (RR 1.17, 95% CI 1.01–1.35), and in (g, g, f, f, f) (RR 1.14, 95% CI 1.01–1.29). As seen in Fig. 1a, there was an increasing trend along the sequences from (0, 0, 0, 0, g) to (g, g, f, f, f), though non-significantly with p-value equal to 0.085.

**Table 3:** Colorectal cancer (CRC) incidence between the age of 60 and 69 by individual invitation sequence to FOBT-screening of the 60–69-years-old population, and compared to control sequence between 60 and 69 (non-invited).

Invitation sequence^a^	No. of individuals (%)	Person-years	No. of CRC	Incidence rate per 100,000 yrs	Rate ratio	95% confidence interval
(0, 0, 0, 0, 0)	15.68	497,599.06	547	109.93	1.00	N/A
(0, 0, 0, 0, g)	19.23	610,517.45	690	113.02	1.03	0.92–1.15
(0, 0, 0, g, g)	13.76	433,112.89	497	114.75	1.04	0.92–1.18
(0, 0, g, g, g)	7.57	237,263.84	277	116.75	1.06	0.92–1.23
(0, g, g, g, g)	7.74	237,333.12	325	136.94	1.25	1.09–1.43
(0, 0, g, g, f)	7.51	233,822.35	283	121.03	1.10	0.96–1.27
(g, g, g, g, f)	7.26	220,277.82	283	128.47	1.17	1.01–1.35
(g, g, g, f, f)	7.29	221,424.82	258	116.52	1.06	0.91–1.23
(g, g, f, f, f)	13.98	386,548.07	487	125.99	1.14	1.01–1.29

^a^0 = no invitation; g = invitation to guaiac-based fecal occult blood test (gFOBT); f = invitation to fecal immunochemistry test (FIT).

When follow-up started at age 70 (Table 4), *i.e.*, when screening invitations had stopped (post screening), the largest decrease in CRC incidence rate, as compared to the non-invited, was seen in the sequences (g, g, g, g, f) and (g, g, g, f, f), RR 0.65, 95% CI 0.47–0.90 and RR 0.53, 95% CI 0.30–0.94, respectively. In the prevalence screening sequence (0, 0, 0, 0, g) there was no increase in the CRC incidence rate, RR 0.93, 95% CI 0.80–1.08, as compared to the non-invited. As seen in Fig. 1b, there was a significant decreasing trend along the sequences from (0, 0, 0, 0, g) to (g, g, f, f, f) with p-value < 0.001.

**Table 4:** Colorectal cancer (CRC) incidence between the age of 70 and 73 by individual invitation sequence to FOBT-screening at 60–69 years, and compared to control sequence between 70 and 73 (non-invited).

Invitation sequence^a^	No. of individuals (%)	Person-years	No. of CRC	Incidence rate per 100,000 yrs	Rate ratio	95% confidence interval
(0, 0, 0, 0, 0)	18.79	140,728.86	296	210.33	1.00	N/A
(0, 0, 0, 0, g)	23.07	226,206.56	442	195.40	0.93	0.80–1.08
(0, 0, 0, g, g)	16.05	120,507.10	240	199.16	0.95	0.80–1.12
(0, 0, g, g, g)	8.74	65,610.40	101	153.94	0.73	0.58–0.92
(0, g, g, g, g)	8.65	64,957.60	112	172.42	0.82	0.66–1.02
(0, 0, g, g, f)	8.59	54,484.00	82	150.50	0.72	0.56–0.92
(g, g, g, g, f)	8.03	30,832.61	42	136.22	0.65	0.47–0.90
(g, g, g, f, f)	8.08	10,799.14	12	111.12	0.53	0.30–0.94

^a^0 = no invitation; g = invitation to guaiac-based fecal occult blood test (gFOBT); f = invitation to fecal immunochemistry test (FIT).

The results of the sensitivity analysis were similar and are presented in Tables 6 and 7 for the screening period and the post screening period, respectively.

**Fig. 2:**
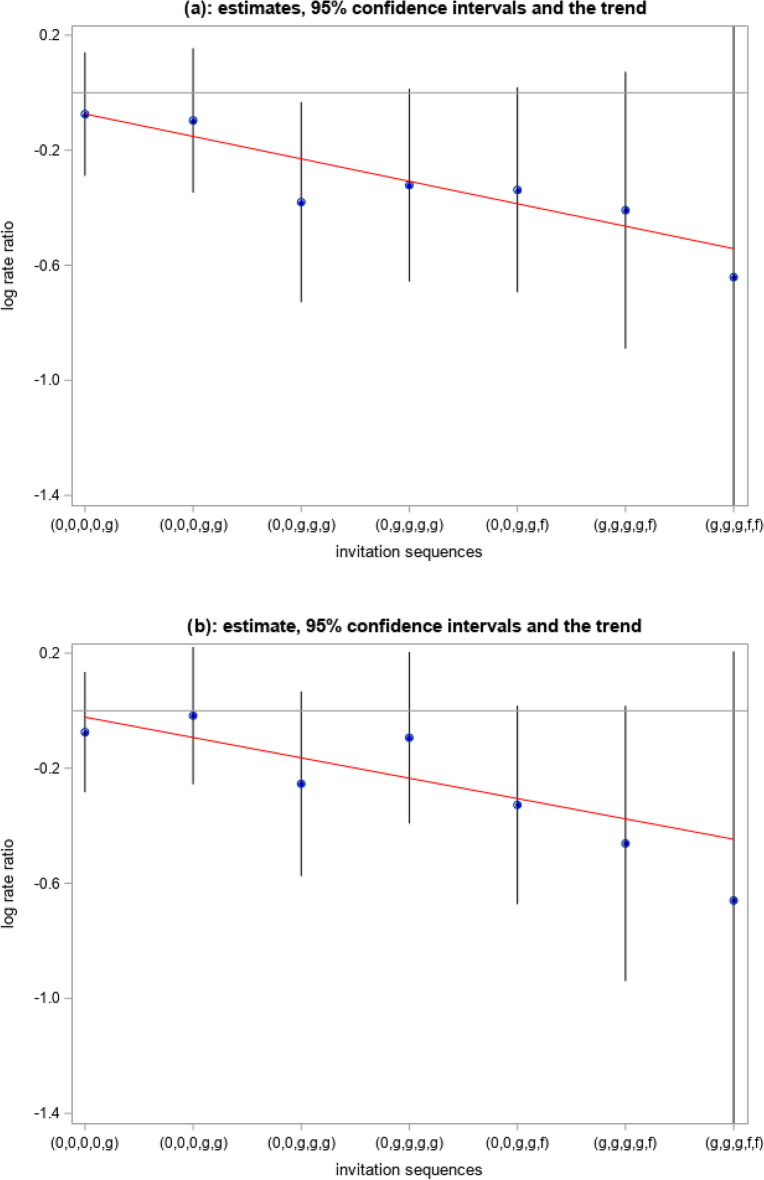
Trend for CRC stage by individual sequence of invitation to FOBT-screening of the 60–69-year-old population relative to control sequence (non-invited). (**a**) Stage 1–2, follow-up between the age of 70 and 73, p-value for trend < 0.001. (**b**) Stage 3–4, follow-up between 70 and 73, p-value for trend < 0.001.

Regarding CRC stages during the post screening period (Table 5), only the invitation sequence (0, 0, g, g, g) had a significantly lower rate of early stages I-II, as compared with the non-invited (RR 0.68, 95% CI 0.48–0.97). As seen in Fig. 2a, b, there was a significant decreasing trend along the sequences from (0, 0, 0, 0, g) to (g, g, f, f, f) with p-value < 0.001 for both stages I-II and III-IV.

**Table 5:** Colorectal cancer (CRC) stage between age 70 and 73 years, by individual invitation sequence to FOBT-screening of the 60–69-years-old population, and compared to control sequence between 70–73 (non-invited).

Invitation sequence^a^	Person-years	Stage^b^	No. of stages	Incidence rate per 100,000 yrs	Rate ratio compared to control^c^	95% confidence interval for rate ratio
(0, 0, 0, 0, 0)	140,728.86	I-II III-IV Missing	134	95.22	ref	
			139	98.77	ref	
			23	16.34		
(0, 0, 0, 0, g)	226,206.56	I-II III-IV Missing	199	87.97	0.93	0.75–1.15
			207	97.51	0.93	0.75–1.14
			36	15.91		
(0, 0, 0, g, g)	120,507.10	I-II III-IV Missing	103	85.47	0.91	0.71–1.18
			117	97.09	0.98	0.77–1.25
			20	16.60		
(0, 0, g, g, g)	65,610.40	I-II III-IV Missing	41	62.49	0.68	0.48–0.97
			51	77.73	0.78	0.56–1.07
			9	13.71		
(0, g, g, g, g)	64,957.60	I-II III-IV Missing	40	61.58	0.73	0.52–1.01
			54	83.13	0.91	0.68–1.28
			18	27.71		
(0, 0, g, g, f)	54,484.00	I-II III-IV Missing	36	66.07	0.71	0.50–1.02
			38	69.75	0.72	0.51–1.02
			8	14.69		
(g, g, g, g, f)	30,832.61	I-II III-IV Missing	18	58.38	0.66	0.41–1.08
			18	58.38	0.63	0.39–1.02
			6	19.46		
(g, g, g, f, f)	10,799.14	I-II III-IV Missing	5	46.30	0.53	0.22–1.27
			5	46.30	0.52	0.22–1.27
			2	18.52		

^a^0 = no invitation; g = invitation to guaiac-based fecal occult blood test (gFOBT); f = invitation to fecal immunochemistry test (FIT).

^b^Stage = I-II for stage I or II; stage = III-IV for stage III or IV; Stage = missing for missing stage.

^c^Rate ratio and 95% CI are estimated after multiple imputing the missing stages.

## Discussion

To our knowledge, this is the first study of a routine FOBT screening program analyzing the effect of different sequential invitation schemes on CRC incidence and stage. Due to the random assignment of invitation sequences, we were able to conduct a sequential analysis unbiased by confounders.

The analysis estimated the effect of the screening program on CRC incidence and stage during different follow-up periods, both during the screening invitations and after screening cessation. During the screening, we found a significantly higher CRC incidence in three of the screening invitation sequences as compared to the non-invited, and post screening the incidence decreased with the number of gFOBT and FIT invitations. Our results are in line with a recent, descriptive age-standardized analysis from the Stockholm-Gotland program comparing four invitation periods; non-invitation, invitation to gFOBT, invitation to FIT, and post invitation^15^, and with another Swedish study that found a significant CRC incidence reduction both during and after screening in the 70–74-year-old population. The latter study, however, relied on a synthetic control group, constructed from data from the regions in Sweden without routine screening^16^.

The benefit of the CRC screening could be seen during the post screening period, *i.e.*, age period 70–73. The CRC incidence decrease was proportional to the number of screening-round invitations and when adding FIT, *e.g.*, with 35% with one round of FIT and 47% with two rounds of FIT. The European Union recommends using FIT in population-based CRC screening, and most countries have adopted this strategy, although the cut-off level for a positive test differ^7,8^. FIT generates a higher compliance when used in CRC screening as compared to gFOBT, *e.g.*, in the Stockholm-Gotland program the participation rate increased from 56.5 to 68.4% when changing to FIT in 2015^17^, and also has a higher sensitivity for premalignant adenomas, *i.e.*, generating higher rates of polypectomies after a positive test^3^. This could affect the incidence of early stage CRC post screening. We demonstrated a decreased trend in the incidence of early-stage CRC with increasing numbers of invitations and with FIT sequences, although the individual FIT sequences did not reach statistical significance in our study. Furthermore, a significant trend was observed for the effectiveness of the screening program on reducing stages III-IV along the sequences from (0, 0, 0, 0, g) to (g, g, g, f, f) post screening. The declining trend in late-staged CRC with increased rounds of screening invitations, could possibly explain the CRC mortality reduction seen in population-based CRC screening^2^.

The CRC incidence increased in all the sequences of invitations during the screening period 60–69 years, as compared to no invitation, although not statistically significant for all sequences. Several studies have demonstrated an increased CRC detection when introducing population based FOBT screening with FIT. In a retrospective study of CRC in the Netherlands, the incidence increased in the population 55 years or older after the introduction of a screening program, but returned below pre-screening levels during follow-up^18^. A similar trend was seen in Italy, with CRC rates peaking during the prevalence rounds of screening, but with a reduction below pre-screening within 5 years^19^. In Denmark, the introduction of population-based FIT-screening nearly doubled the incidence of CRCs, as compared to the not yet invited^20^. On the contrary, the present study did not show an increased detection of CRC for the sequence (0, 0, 0, 0, g). However, this sequence corresponds to the prevalence screening round of a gFOBT program starting at age 68. Moreover, the aforementioned studies were largely empirical as compared to our sequential analysis of invitations, CRC incidence and stage on individual data.

Regarding the decrease in CRC incidence with gFOBT in population-based screening, one older randomized study from Minnesota, U.S.A., has demonstrated a CRC incidence reduction of 20% and 17%, with annual and biennial screening, respectively, after 18 years of follow-up and with 6 and 11 rounds of screening being offered^21^. However, our sequential analysis demonstrated a much larger decrease of the CRC incidence in the population-based screening program up to 3 years after screening, possibly because the effectiveness of screening is likely to diminish with time after screening cessation (no longer exposed to screening), and because of the addition of FIT-rounds. The gradient of the decrease was proportional to the number of screening rounds and particularly with FIT in the sequence of invitation. Moreover, the Minnesota trial included up to 80-year-olds with a higher CRC incidence than the 60–69-year-olds invited or not invited in the present study.

The strength of our study was our study design, *i.e.*, the random assignment of invitation sequences by birth years, which well mimics a randomized trial of invitation sequences on the population level. The study design avoided a complex—often unreliable—adjustment of time-dependent confounding factors. Another strength was our high-quality individual-based massive data collected in the same region during close calendar years over a 10-year screening period and a 3-year post-screening period. With our study design and data, this study revealed the dynamic of CRC incidences and stage during the screening and post-screening periods of the complex screening process, which could serve as a base for comprehensive predictive modeling and individualized screening programs according to CRC risk.

This study has limitations. Firstly, as a trade-off for less condounding influences, we estimated the effects of screening sequences rather than the effects of individual screening rounds. However, it is interesting to see the effects of individual invitation rounds, particularly with FIT. This will be achieved by a more comprehensive predictive modeling. Secondly, our study had a short follow-up, only 3 years post screening, and hence, did not address the long-term effectiveness of the routine screening program in decreasing CRC incidence. Thirdly, due to our limited sequences, we could not adjust for the calendar year—the only potential confounder in our study design. Given the fact that our follow-up covered only a relatively short calendar period, the calendar-year influence is typically small at least for CRC cancer. As a compromise, we only assessed the assumption by sensitivity analysis.

## Conclusion

In conclusion, this analysis of different sequences of invitation to the routine FOBT screening of Stockholm-Gotland, Sweden, revealed an increased CRC detection during screening for three of the sequences and a decreased incidence post screening that was proportional to the number of invitations to gFOBT and FIT rounds. The decreasing trend was seen for both early and late staged CRC. This has implications for future modeling studies and risk-based screening strategies and could partly explain the reduction in CRC mortality reported from the program.

## Supplementary Information

Below is the link to the electronic supplementary material.

Supplementary Information.

## Data Availability

The original pseudonymized data is not supplemented, but could be available for research upon request to Principal Investigator Johannes Blom and within the framework of the Swedish data protection legislation and any required permissions from authorities.

## Appendix

See Tables

**Table 6:** Incidence of colorectal cancer (CRC) between age 60 and 69 years, by individual invitation sequence to FOBT-screening of the 60–69-years-old population, compared to control sequence between 60–69 (non-invited), using the rates at different calendar years for CRC in the Swedish population between 60 and 69 as reference.

Invitation sequence^a^	No. of individuals (%)	Person-years	No. of CRC	CRC incidence	Rate ratio of CRC	95% confidence interval
(0, 0, 0, 0, 0)	15.68	497,599.06	547	109.93	1.00	N/A
(0, 0, 0, 0, g)	19.23	610,517.45	690	113.02	1.03	0.92–1.15
(0, 0, 0, g, g)	13.76	433,112.89	497	114.75	1.04	0.92–1.18
(0, 0, g, g, g)	7.57	237,263.84	277	116.75	1.07	0.92–1.25
(0, g, g, g, g)	7.74	237,333.12	325	136.94	1.26	1.08–1.46
(0, 0, g, g, f)	7.51	233,822.35	283	121.03	1.10	0.96–1.27
(g, g, g, g, f)	7.26	220,277.82	283	128.47	1.18	1.01–1.37
(g, g, g, f, f)	7.29	221,424.82	258	116.52	1.07	0.90–1.26
(g, g, f, f, f)	13.98	386,548.07	487	125.99	1.15	1.01–1.30

^a^0 = no invitation; g = invitation to guaiac-based fecal occult blood test (gFOBT); f = invitation to fecal immunochemistry test (FIT).

6 and

**Table 7:** Incidence of colorectal cancer (CRC) between age 70 and 73 years, by individual invitation sequence to FOBT-screening of the 60–69-years-old population, compared to control sequence between 70 and 73 (non-invited), using the rates at different calendar years for CRC in the Swedish population between 70 and 74 as reference.

Invitation sequence^a^	No. of individuals (%)	Person-years	No. of CRC	CRC incidence	Rate ratio of CRC	95% confidence interval
(0, 0, 0, 0, 0)	18.79	140,728.86	296	210.33	1.00	N/A
(0, 0, 0, 0, g)	23.07	226,206.56	442	195.40	0.89	0.77–1.04
(0, 0, 0, g, g)	16.05	120,507.10	240	199.16	0.77	0.61–0.97
(0, 0, g, g, g)	8.74	65,610.40	101	153.94	0.69	0.55–0.87
(0, g, g, g, g)	8.65	64,957.60	112	172.42	0.72	0.57–0.91
(0, 0, g, g, f)	8.59	54,484.00	82	150.50	0.55	0.41–0.75
(g, g, g, g, f)	8.03	30,832.61	42	136.22	0.49	0.34–0.72
(g, g, g, f, f)	8.08	10,799.14	12	111.12	0.41	0.23–0.76

^a^0 = no invitation; g = invitation to guaiac-based fecal occult blood test (gFOBT); f = invitation to fecal immunochemistry test (FIT).

7.
